# Synthesis, physiochemical property and antimicrobial activity of novel quaternary ammonium salts

**DOI:** 10.1080/14756366.2017.1396456

**Published:** 2017-11-17

**Authors:** Xianrui Xie, Wei Cong, Feng Zhao, Hongjuan Li, Wenyu Xin, Guige Hou, Chunhua Wang

**Affiliations:** School of Pharmacy, the Key Laboratory of Prescription Effect and Clinical Evaluation of State Administration of Traditional Chinese Medicine of China, Binzhou Medical University, Yantai, P. R. China

**Keywords:** 5-Phenyl-1,3,4-oxadiazole-2-thiol, quaternary ammonium salts, antibacterial activity, antifungal activity, cytotoxicity

## Abstract

Twenty-four novel 5-phenyl-1,3,4-oxadiazole-2-thiol (POT) analogues, benzo[*d*]oxazole-2-thiol, benzo[*d*]thiazole-2-thiol and 5-methyl-1,3,4-thiadiazole-2-thiol-substituted *N,N*-bis(2-hydroxyethyl) quaternary ammonium salts (QAS) (**5a-d, 6a-d, 7a-d, 10a-d, 13a-d, 16a-d**) were prepared and characterised by FTIR, NMR and elemental analysis. Part of target compounds (**5d**, **6d**, **7d**, **10d**, **13d**, **16d**) displayed potent antimicrobial effect against ten common pathogens (*S. aureus, α-H-tococcus, β-H-tococcus, E. coli, P. aeruginosa, Proteus vulgaris, Canidia Albicans, Cytospora mandshurica, Physalospora piricola, Aspergillus niger*) and had relatively low cytotoxity against two human cell lines (HaCat and LO2). TEM and SEM images of *E. coli* and *S. aureus* morphologies treated with **7d** showed that the antibacterial mechanism might be the QAS fixing on cell wall surfaces and puncturing to result in the release of bacterial cytoplasm. This study provides new information of QAS, which could be used to design novel antimicrobial agents applied in clinic or agriculture.

## Graphical abstract

**Figure:**
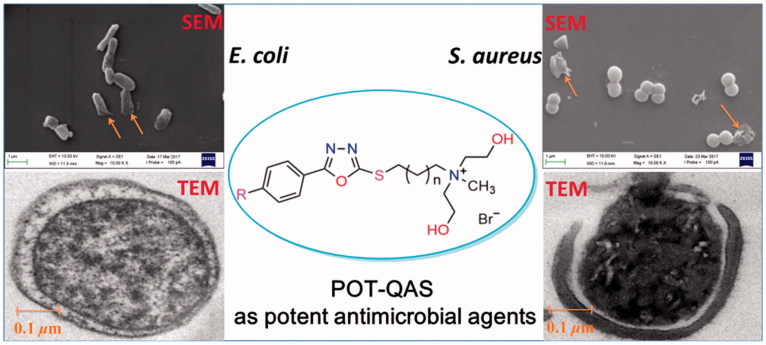

## Introduction

As one of the leading causes of death worldwide, outbreaks of infectious diseases triggered by bacteria, viruses and fungi lead to over one-fourth of global deaths annually^1^ ^,^ ^2^. Thus, there is an urgent demand for exploring more efficient, broad-spectrum and long-lasting antimicrobial agents. Because of excellent antibacterial activities, quaternary ammonium salts (QAS) are widely used by the pharmaceutical industry for their recognised activity against fungi, bacteria, viruses and parasites^3^. For example, QASs are used as antiseptics and disinfectants of medical apparatus and instruments, and a variety of clinical application, such as preoperative disinfection of unbroken skin and trauma^4–9^. With more and more resistant organisms continue to emerge on clinic, the ideal antimicrobial agents should have to possess strong antibacterial activity, be safe in relation to humans and should not persist in the environment for a long time^10^. Some of the quaternary ammonium compounds fulfil these conditions have been invented and received increasing attentions as antibacterial agents^11–13^. Especially, long-chain alkyl-QAS compounds show unsurpassed antibacterial effects. It is well-known that the antibacterial activity and drug toxicity are influenced by the alkyl chain length of these agents^14^ ^,^ ^15^. As the growth of the alkyl chains, the antibacterial activities gradually increase, until reaching a reasonable limit. Unfortunately, drug toxicity of QAS bearing longer-chain alkyl groups also strengthen as well as benzyl-QAS. Tremendous research efforts have been devoted to development of alternative antibacterial therapeutics that are not easily forming resistance, such as quaternary ammonium salts (QAS), which destruct cell membrane of bacteria and induce the leakage of intracellular components from bacterial cells^16^. Thus, it becomes urgent to design and prepare one kind of novel antibacterial with higher antibacterial activities and lower drug toxicity. Many discrete QAS^17^ and polymerised quaternised materials (such as quaternised chitosan^18^ ^,^ ^19^) were reported with good antibacterial activities because of quick disinfection and sterilisation.

**Figure 1.**
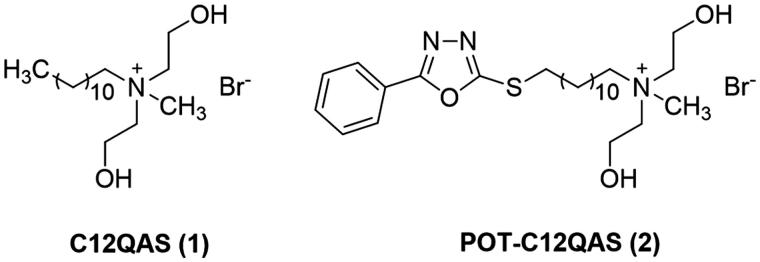
Representative target compounds in previous work.

In the previous work of our group, *N*-methyl-*N*-R-*N*,*N*-bis(2-hydroxyethyl) ammonium bromides (BNQAS, C12QAS, C14QAS, C16QAS, C18QAS) were prepared from *N*-methyldiethanolamine and halohydrocarbon with long-chain alkyl bromides^20^. These QASs showed good antibacterial abilities against *E. coli, S. aureus*, *B. subtilis* and antifungal activities against *Cytospora mandshurica, Botryosphaeria ribis, Physalospora piricola and Glomerella cingulata*. The strategy provided a facile way to design and develop new types of antimicrobial agents for application in preventing the fruit rot, especially apple. Meanwhile, these QASs also were used to prepare nontoxic *O*-quaternised antimicrobial chitosan materials^21^. Simultaneously, C12QAS (**1**, Figure 1) with the best antibacterial abilities was also used to prepare novel block antibacterial polyurethane (BAPU) and terminated antibacterial polyurethane (TAPU) through prepolymerisation reaction^19^. However, QAS monomers display higher cytotoxicity towards normal cell lines^20^, which may limit their clinical utility as antibacterial material. So it is a big challenge that how to improve their antimicrobial activities on the basis of lower cytotoxicity.

Motivated by our interest in the antimicrobial agents, chemical modifications of QAS monomers were carried out immediately. Due to the quaternary ammonium group was a key active group, *N,N*-bis(2-hydroxyethyl) ammonium bromide of C12QAS (**1**, Figure 1) was retained. Studied through literature researches, we found many 1,3,4-oxadiazole-2-thiol derivatives were reported as potent inhibitors of Gram-negative and Gram-positive bacteria^22–25^. Very recently, we tried to change long-chain alkyl group of C12QAS (**1**, Figure 1) into 5-phenyl-1,3,4-oxadiazole-2-thiol (POT)^26^ substituted alkyl groups and synthesised dozens of POT-QASs^27^. Among these, bearing *N*,*N*-bis(2-hydroxyethyl) and POT groups, POT-C12QAS (**2**, Figure 1) displayed unsurpassed antimicrobial activities and low cytotoxicity. The structure–activity relationships indicated that POT and flexible dihydroxyethyl group in QAS were necessary for antibacterial activities.

**Scheme 1.**
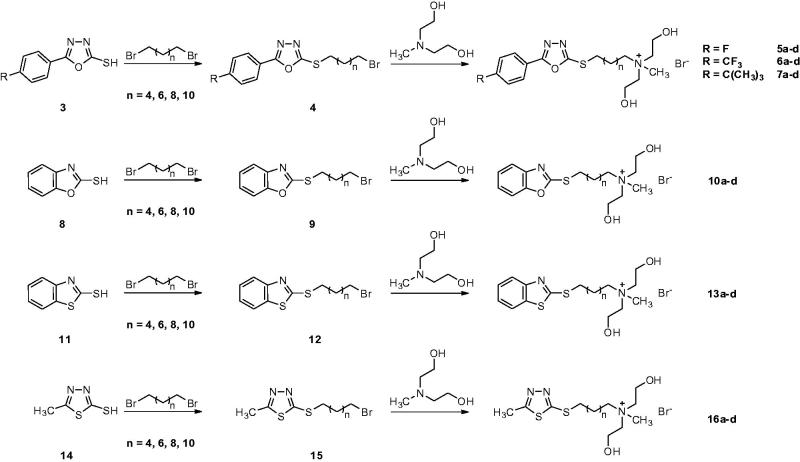
The synthesis and structures of six series of noval QASs.

Furthermore, in order to improve antimicrobial activities and decrease cytotoxicity, we tried to modify or replace the POT fragment in POT-QAS with 5-(4-fluorophenyl)-1,3,4-oxadiazole-2-thiol (POTF), 5-(4-trifluoromethyl phenyl)-1,3,4-oxadiazole-2-thiol (POTT), 5-(4-*tert*-butylphenyl)-1,3,4-oxadiazole-2-thiol (POTB), benzo[*d*]oxazole-2-thiol, benzo[*d*]thiazole-2-thiol and 5-methyl-1,3,4-thiadiazole-2-thiol fragments. Based on the consideration, six series of novel QASs (**5a-d, 6a-d, 7a-d, 10a-d, 13a-d, 16a-d**, Scheme 1) were prepared. These novel QASs were characterised with proton nuclear magnetic resonance (^1^H NMR and ^13^C NMR) pectroscopy, stransmission Fourier transform infrared (FTIR) spectra, elemental analyses and melting point. Their antibacterial, antifungal properties and cytotoxicity were tested and the antibacterial mechanism of quaternary ammonium salts to *E. coli* and *S. aureus* was explored by SEM and TEM.

## Results and discussion

### Structural analysis

In this study, 24 5-phenyl-1,3,4-oxadiazole-2-thiol (POT) analogues, benzo[*d*]oxazole-2-thiol, benzo[*d*]thiazole-2-thiol and 5-methyl-1,3,4-thiadiazole-2-thiol substituted *N*,*N*-bis(2-hydroxyethyl) quaternary ammonium salts (**5a-d, 6a-d, 7a-d, 10a-d, 13a-d, 16a-d**) were prepared from Aromatic thiols, α,ω-dibromoalkanes and *N*-methyldiethanolamine in good yields (Scheme 1). Their structures were characterised by FTIR, NMR and elemental analysis. From the FTIR spectra, strong characteristic absorption band of hydroxyl and stretching vibration band of C-O bonds can be found. For example, the two bands of **5a** lie in 3307 cm^−1^ and 1054 cm^−1^, respectively, which demonstrates the existence of –OH of QAS. In addition, the distinct band in 788 cm^−1^ belongs to the stretching vibration band of linear paraffin in FTIR spectra of **5a**. In other QAS, the stretching vibration bands of linear paraffin are 731 cm^−1^ (**6a**), 739 cm^−1^ (**7a**), 756 cm^−1^ (**10a**), 741 cm^−1^ (**13a**) and 729 cm^−1^ (**16a**), respectively. In ^1^H NMR spectra, the single peak of hydroxy of **5a** can be found at *δ* 5.31 ppm, the multiple peaks of benzene ring disappeared in ^1^H NMR spectra at *δ* 8.16–7.22 ppm, and the single peaks of methyl of **5a** at *δ* 3.06 ppm. The similar structure characteristic also can prove their structure for other compounds and they are shown in experimental part. Additionally, ^13^C NMR, element analysis further confirms the correctness of their structures.

### Antimicrobial properties

In this paper, antimicrobial activities of 24 QASs were detected by quantitative tests. The data of minimal inhibitory concentration (MIC) and minimum bactericidal concentration (MBC) for 24 QASs against Gram-positive bacteria (*S. aureus*, *α-H-tococcus*, *β-H-tococcus*), Gram-negative bacteria (*E. coli*, *P. aeruginosa*, *Proteus vulgaris*) and fungi (*Canidia albicans*, *Cytospora mandshurica*, *Physalospora piricola*, *Aspergillus niger*) were obtained as shown in Tables 1 and 2, respectively. Benzalkonium chloride (BZK) and chlorhexidine acetate (CA) was selected as positive controls. Representative compound POT-C12QAS (**2**) which synthesised and evaluated in previous work was also taken as a reference compound in MIC, MBC and cytotoxicity tests.

**Table 1.** Minimal inhibitory concentration (MIC) of QASs (μg/mL).

Compd	*S. aureus*	*α-H-tococcus*	*β-H-tococcus*	*E. coli*	*P. aeruginosa*	*Proteus vulgaris*	*Canidia albicans*	*Cytospora mandshurica*	*Physalospora piricola*	*Aspergillus niger*
**BZK**	3.125	3.125	1.56	25	25	25	12.5	6.25	3.125	3.125
**CA**	3.125	6.25	1.56	6.25	25	12.5	12.5	6.25	1.56	3.125
**2**	6.25	6.25	6.25	25	12.5	25	6.25	3.125	3.125	3.125
**5a**	100	50	25	100	100	100	50	50	12.5	50
**5b**	25	12.5	12.5	25	50	50	25	25	6.25	50
**5c**	25	12.5	6.25	25	50	50	25	12.5	3.125	25
**5d**	12.5	12.5	6.25	25	50	25	12.5	12.5	3.125	25
**6a**	50	50	25	50	100	100	50	12.5	6.25	25
**6b**	25	12.5	12.5	25	50	50	25	12.5	3.125	12.5
**6c**	25	12.5	6.25	25	50	25	25	12.5	3.125	12.5
**6d**	6.25	6.25	3.125	25	50	25	12.5	6.25	1.56	6.25
**7a**	50	25	12.5	50	50	50	25	12.5	12.5	25
**7b**	25	12.5	6.25	25	50	25	12.5	6.25	3.125	12.5
**7c**	12.5	12.5	3.125	25	50	25	6.25	6.25	1.56	6.25
**7d**	6.25	6.25	1.56	12.5	25	12.5	6.25	3.125	1.56	6.25
**10a**	100	50	25	100	100	100	50	50	25	50
**10b**	50	25	12.5	50	50	50	50	25	12.5	25
**10c**	25	25	6.25	50	50	50	25	6.25	6.25	12.5
**10d**	12.5	12.5	6.25	25	25	25	12.5	6.25	3.125	6.25
**13a**	50	50	12.5	50	100	100	25	25	25	25
**13b**	25	25	12.5	50	50	50	25	6.25	6.25	12.5
**13c**	12.5	25	6.25	25	25	25	12.5	3.125	3.125	6.25
**13d**	6.25	12.5	1.56	25	25	25	6.25	1.56	3.125	3.125
**16a**	100	50	50	100	200	100	50	50	25	50
**16b**	50	50	25	50	100	50	25	25	12.5	25
**16c**	25	25	6.25	50	50	50	25	12.5	12.5	12.5
**16d**	12.5	12.5	6.25	25	50	25	12.5	12.5	6.25	12.5

**Table 2.** Minimal bactericidal concentration (MBC) of QASs (μg/mL).

Compd	*S. aureus*	*α-H-tococcus*	*β-H-tococcus*	*E. coli*	*P. aeruginosa*	*Proteus vulgaris*	*Canidia albicans*	*Cytospora mandshurica*	*Physalospora piricola*	*Aspergillus niger*
**BZK**	3.125	3.125	1.56	25	25	25	25	6.25	3.125	6.25
**CA**	6.25	6.25	3.125	12.5	25	12.5	12.5	6.25	3.125	6.25
**2**	12.5	6.25	6.25	50	25	50	12.5	6.25	3.125	3.125
**5a**	100	50	25	100	100	100	50	50	12.5	50
**5b**	50	25	12.5	50	50	50	25	25	6.25	50
**5c**	25	12.5	12.5	50	50	50	25	12.5	6.25	25
**5d**	12.5	12.5	6.25	25	50	25	12.5	12.5	3.125	25
**6a**	50	50	25	50	100	100	50	25	12.5	25
**6b**	25	25	12.5	50	50	50	25	12.5	6.25	25
**6c**	25	12.5	6.25	25	50	25	25	12.5	3.125	12.5
**6d**	12.5	6.25	3.125	25	50	25	12.5	6.25	1.56	6.25
**7a**	50	25	12.5	50	50	50	25	12.5	12.5	25
**7b**	25	12.5	6.25	25	50	25	12.5	12.5	3.125	12.5
**7c**	12.5	12.5	6.25	25	50	25	12.5	6.25	3.125	12.5
**7d**	6.25	6.25	1.56	12.5	25	12.5	6.25	3.125	1.56	6.25
**10a**	100	50	25	100	200	100	100	50	25	50
**10b**	50	50	25	100	100	50	50	25	25	25
**10c**	25	25	12.5	50	50	50	25	12.5	6.25	12.5
**10d**	12.5	12.5	6.25	25	25	25	12.5	6.25	6.25	12.5
**13a**	50	50	25	100	100	100	50	25	25	25
**13b**	50	25	12.5	50	100	50	25	12.5	12.5	12.5
**13c**	12.5	25	6.25	25	50	25	25	6.25	3.125	6.25
**13d**	6.25	12.5	3.125	25	25	25	6.25	6.25	3.125	6.25
**16a**	200	100	50	200	200	200	50	50	25	50
**16b**	100	50	25	100	100	50	50	25	25	25
**16c**	25	25	12.5	50	50	50	25	25	12.5	25
**16d**	25	12.5	6.25	25	50	25	12.5	12.5	6.25	12.5

For the six series of QASs (**5a-d**, **6a-d**, **7a-d**, **10a-d**, **13a-d**, **16a-d),** all of their structures contained nitrogen-containing heterocyclics and *N*,*N*-bis(2-hydroxyethyl) quaternary ammonium salt pharmacophores linked by medium- or long-chain alkyl groups (hexyl, octyl, decyl and dodecyl). Compared with our previous research, similar result was found that their MIC and MBC values decreased in turn following the increase of chain length. In other words, compounds **5d**, **6d**, **7d**, **10d**, **13d** and **16d** could regard as the most potent compounds in each series.

Although compounds **5a-d**, **6a-d** and **7a-d** belonged to POT-QAS derivatives, they exhibited diverse antibacterial activities. For Gram-positive bacteria (*S. aureus*, *α-H-tococcus*, *β-H-tococcus*) and Gram-negative bacteria (*E. coli*, *P. aeruginosa*, *Proteus vulgaris*), compound **7d** bearing with 5-(4-*tert*-butylphenyl)-1,3,4-oxadiazole-2-thiol (POTB) substituent group exhibited the best antibacterial activities among all six series (corresponding MIC values, Table 2). It is just similar to unmodified POT-C12QAS: ca. 6.25 μg/mL against *S. aureus*, *α-H-tococcus* ca. 1.56 μg/mL against *β-H-tococcus*; ca. 12.5 μg/mL against *E. coli*, *Proteus vulgaris*, ca. 25 μg/mL against *P. aeruginosa*, Table 1; MBC values were equal to corresponding MIC values, Table 2. It should be pointed out that the antibacterial activities of **7d** were comparable to positive controls (**BZK** and **CA**), and more potent than unmodified POT-C12QAS (**2**). Furthermore, Introduced halogen substitution, especially fluoro substitution into the structure was hopeful to enhance the antibacterial effect^28^ ^,^ ^29^. But to our disappointment, fluoro (**5d**) or trifluoromethyl (**6d**) substituted derivatives did not exhibit desired antibacterial activities, and their MIC and MBC values only reached to medium level (e.g. MIC values for **5d**: ca. 12.5 μg/mL against *S. aureus*, *α-H-tococcus*, ca. 6.25 μg/mL against *β-H-tococcus*; ca. 25 μg/mL against *E. coli*, *Proteus vulgaris*, ca. 50 μg/mL against *P. aeruginosa*, Table 1). These foregoing results suggest that electron-donating substituents (tertiary butyl) on POT might enhance antibacterial activities of POT-QAS; on the contrary, electron-withdrawing substituents (fluoro or trifluoromethyl) might give little contribution to the activities.

In order to investigate the influence of other nitrogen heterocyclics substituted in QAS, POT fragment was changed into benzo[*d*]oxazole-2-thiol (**10a-d**), benzo[*d*]thiazole-2-thiol (**13a-d**) or 5-methyl-1,3,4-thiadiazole-2-thiol (**16a-d**), respectively. Among these three non-POT series, compound **13d** exhibited relatively potent antibacterial activities (MIC: ca. 6.25 μg/mL against *S. aureus*, ca. 12.5 μg/mL against *α-H-tococcus*, ca. 1.56 μg/mL against *β-H-tococcus*; ca. 25 μg/mL against *E. coli*, *Proteus vulgaris*, *P. aeruginosa*, Table 1; MBC: ca. 3.125 μg/mL against *β-H-tococcus*, other MBC values were equal to corresponding MIC values, Table 2), which was close to positive controls (**BZK** and **CA**). However, besides compound **10d** possessed fairly activities against Gram-negative bacteria (MIC: ca. 25 μg/mL against *E. coli*, *Proteus vulgaris*, *P. aeruginosa*, Table 1; MBC values were equal to corresponding MIC values, Table 2), other non-POT derivatives did not exhibit noticeable activities. Based on the above results, POT-QAS derivatives were partially superior to non-POT-QAS derivatives, and this might be because POT-QAS derivatives with flexible alkyl chain length and POT fragment could more easily pass through cell membrane into the cell to damage bacteria^20^ ^,^ ^27^ ^,^ ^30–32^.

In the antifungal activity assay of the target compounds, four strains of common fungi in clinical practice and agriculture (*Canidia albicans*, *Cytospora mandshurica*, *Physalospora piricola*, *Aspergillus niger*) were tanken as the target fungi. As shown in Tables 1 and 2, to our delight, most compounds bearing with the long-chain alkyl group (dodecyl, e.g. **5d**, **6d**, **7d**, **10d**, **13d** and **16d**) displayed fairly good antifungal activities. The most noticeable compound was **13d** (MIC: ca. 6.25 μg/mL against *Canidia albicans*, ca. 1.56 μg/mL against *Cytospora mandshurica*, ca. 3.125 μg/mL against *Physalospora piricola*, *Aspergillus niger*, Table 1; MBC: ca. 6.25 μg/mL against *Cytospora mandshurica*, other MBC values were equal to corresponding MIC values, Table 2), with a 2-mercaptobenzothiazole substituent in C12QAS, exhibited more potent activities than positive controls (**BZK** and **CA**). In addition, POT-C12QAS (**2**) derivative **7d** (MIC: ca. 6.25 μg/mL against *Canidia albicans*, ca. 3.125 μg/mL against *Cytospora mandshurica*, ca. 1.56 μg/mL against *Physalospora piricola*, ca. 6.25 μg/mL against *Aspergillus niger*, Table 1; MBC values were equal to corresponding MIC values, Table 2) also displayed almost the same antifungal activities as positive controls (**BZK** and **CA**). These encouraging results indicated that either POTT (**7d**) or 2-mercaptobenzothiazole (**13d**) substituent in C12QAS could enhance the antifungal activities. More than that, both of which might develop into promising antifungal agents in clinic or agriculture.

### Cytotoxicity

These six series of antimicrobial compounds may be applied to daily or clinical disinfectants for unbroken skin and trauma, or agricultural fungicides alternatively. Thus, their cytotoxicity for skin or body tissues are crucial. In this study, the cytotoxicity of target compounds was evaluated for human immortalised epidermal (HaCat) and human normal liver (LO2) cell line using MTT assay. The results were showed in Table 3 summarising the corresponding IC_50_ values of QASs. The IC_50_ values of **5a**, **6a**, **10a**, **16a**, **16 b** were about more than 50 μg/mL for LO2 and HaCat. In addition, compounds **5 b**, **6 b**, **10 b**, **13a**, **16c**, **16d** also showed lower IC_50_ values (more than 25 μg/mL). These data demonstrated that compounds **5a**, **6a**, **16a**, **16 b** were non-toxic for LO2 and HaCat. Especially, lower-toxic compounds **5 b**, **6 b**, **16c**, **16d** with better activities might become superior antimicrobial agents. However, the IC_50_ values of each series for LO2 and HaCat decreased gradually and finally reached to 4.16 μg/mL. For example, the IC_50_ values of **7a-d** were orderly 44.26 ± 3.31 μg/mL, 16.78 ± 1.35 μg/mL, 12.90 ± 1.37 μg/mL, 5.08 ± 0.51 μg/mL for LO2 as well as 23.84 ± 2.13 μg/mL, 9.05 ± 0.92 μg/mL, 6.94 ± 0.53 μg/mL,4.16 ± 0.43 μg/mL for HaCat, respectively. These results indicated that their cytotoxicity and antimicrobial activities synchronously increased following the increased of chain length from hexyl group to dodecyl group^27^, and all of them exhibited weaker cytotoxicity compared with positive controls (BZK and CA). In addition, it should be pointed out that compounds **7d** and POT-C12QAS (**2**) with POT and dodecyl group displayed weaker cytotoxicity than *N*-methyl-*N*-dodecyl-*N,N*-bis(2-hydroxyethyl) ammonium bromides (C12QAS), which IC_50_ value was ca. 4.00 μg/mL^20^. This result proved that introduction of POT fragment in C12QAS could effectively reduce cytotoxicity and improve antimicrobial activities.

**Table 3.** Cytotoxicity of QASs for HaCat and LO2 cells.

	IC_50_ (μg/mL)			IC_50_ (μg/mL)			IC_50_ (μg/mL)	
Compd	LO2	HaCat	Compd	LO2	HaCat	Compd	LO2	HaCat
**BZK**	2.12 ± 0.43	3.19 ± 0.26	**CA**	2.06 ± 0.06	2.54 ± 0.12	**2**	14.23 ± 2.15	18.27 ± 2.21
**5a**	83.51 ± 5.67	76.52 ± 4.58	**7a**	44.26 ± 3.31	23.84 ± 2.13	**13a**	64.28 ± 4.12	45.06 ± 3.23
**5b**	46.75 ± 3.32	50.83 ± 3.65	**7b**	16.78 ± 1.35	9.05 ± 0.92	**13b**	29.58 ± 2.86	17.84 ± 1.56
**5c**	23.86 ± 1.68	19.39 ± 1.23	**7c**	12.90 ± 1.37	6.94 ± 0.53	**13c**	16.84 ± 1.61	10.51 ± 1.02
**5d**	6.95 ± 0.62	6.14 ± 0.53	**7d**	5.08 ± 0.51	4.16 ± 0.43	**13d**	8.96 ± 0.46	6.02 ± 0.35
**6a**	74.35 ± 4.12	62.57 ± 3.82	**10a**	88.27 ± 5.89	54.62 ± 3.16	**16a**	>100	>100
**6b**	38.72 ± 2.92	32.57 ± 2.69	**10b**	42.92 ± 2.37	30.38 ± 2.89	**16b**	86.52 ± 5.33	65.38 ± 3.12
**6c**	19.56 ± 1.62	12.06 ± 1.57	**10c**	25.32 ± 1.78	15.86 ± 1.23	**16c**	43.18 ± 2.67	36.87 ± 2.81
**6d**	5.86 ± 0.61	5.25 ± 0.37	**10d**	9.96 ± 0.76	7.14 ± 0.51	**16d**	30.12 ± 2.32	27.03 ± 2.53

### Haemolysis

The surfactant may be absorbed and penetrate to the cell membrane, where it makes osmotic phenomena by altering the permeability of membrane, which in turn causes the cellular lysis^33^. Haemolysis assay gives quantitative estimation about haemoglobin release when red blood cells are treated with QASs. Haemolytic assay of QASs at different concentration was carried out in Table 4. It was observed that the haemolysis rate of these samples increased successively following the increase of concentration, which showed concentration-dependent haemolysis. More importantly, all QASs did not cause haemolysis under the concentration of 50 μg/mL except **10d** and **13d**. For **5a-b, 6a-b, 10a-c, 13a-b, and 16a-d**, their safe concentration can more than 100 μg/mL. Compared with BZK and CA, all QASs were safe to be used when they circulated into the blood in low concentration.

**Table 4.** Haemolysis rate (%) of QASs with different concentration (μg/mL).

	Haemolysis rate (%)						
Compd	6.25	12.50	25.00	50.00	100.00	200.00	400.00
**BZK**	1.51 ± 0.31	1.39 ± 0.38	12.69 ± 1.87	63.85 ± 4.56	92.36 ± 3.78	96.28 ± 2.97	98.53 ± 1.23
**CA**	0.86 ± 0.28	2.51 ± 0.87	5.29 ± 1.22	37.05 ± 3.78	62.18 ± 3.53	86.02 ± 3.82	95.67 ± 3.54
**2**	0.18 ± 0.05	0.42 ± 0.13	0.93 ± 0.41	4.11 ± 0.89	17.07 ± 2.34	82.75 ± 3.18	90.61 ± 4.23
**5a**	0.21 ± 0.03	0.53 ± 0.15	1.37 ± 0.87	1.54 ± 0.96	3.25 ± 1.23	6.21 ± 3.21	35.64 ± 4.87
**5b**	0.29 ± 0.08	0.68 ± 0.23	1.87 ± 1.35	2.19 ± 1.29	3.92 ± 1.02	21.08 ± 4.55	65.49 ± 4.13
**5c**	0.43 ± 0.16	0.75 ± 0.19	2.31 ± 0.67	4.89 ± 0.53	33.13 ± 4.87	52.28 ± 6.34	67.15 ± 5.89
**5d**	0.49 ± 0.12	0.87 ± 0.27	2.43 ± 0.82	4.81 ± 0.74	32.41 ± 6.78	66.71 ± 5.21	66.92 ± 4.12
**6a**	0.15 ± 0.08	0.35 ± 0.17	0.51 ± 0.19	0.99 ± 0.12	3.38 ± 1.23	7.09 ± 2.89	40.96 ± 5.32
**6b**	0.18 ± 0.06	0.51 ± 0.08	0.43 ± 0.10	0.70 ± 0.18	4.61 ± 0.31	26.16 ± 3.68	80.37 ± 4.98
**6c**	0.57 ± 0.15	0.42 ± 0.13	0.67 ± 0.13	3.56 ± 1.32	35.76 ± 5.10	82.52 ± 6.12	89.39 ± 4.35
**6d**	0.64 ± 0.19	1.05 ± 0.28	2.02 ± 3.45	4.85 ± 4.65	36.43 ± 6.35	85.62 ± 4.86	92.46 ± 3.87
**7a**	1.03 ± 0.56	2.50 ± 1.08	1.01 ± 0.58	1.98 ± 0.68	5.66 ± 1.23	20.76 ± 3.28	34.73 ± 4.78
**7b**	1.41 ± 0.43	1.48 ± 0.68	2.20 ± 0.68	2.59 ± 0.89	13.84 ± 2.73	30.63 ± 3.76	85.75 ± 5.67
**7c**	0.18 ± 0.06	0.75 ± 0.19	1.19 ± 0.76	1.92 ± 0.87	14.08 ± 0.69	38.82 ± 3.12	79.03 ± 4.08
**7d**	1.51 ± 0.91	1.71 ± 0.12	2.32 ± 1.86	4.77 ± 1.57	12.54 ± 4.79	39.62 ± 3.93	87.75 ± 3.26
**10a**	0.13 ± 0.06	0.40 ± 0.08	0.43 ± 0.10	0.83 ± 0.43	0.31 ± 0.11	1.27 ± 0.85	1.26 ± 0.82
**10b**	0.17 ± 0.09	0.36 ± 0.09	0.12 ± 0.05	0.09 ± 0.02	0.85 ± 0.32	1.89 ± 0.72	1.52 ± 0.87
**10c**	0.36 ± 0.08	1.37 ± 0.18	1.75 ± 0.53	3.87 ± 1.41	1.89 ± 0.67	34.67 ± 4.91	53.50 ± 6.04
**10d**	1.28 ± 0.87	2.36 ± 1.23	3.31 ± 1.13	6.02 ± 1.11	39.95 ± 3.25	60.77 ± 4.37	74.32 ± 4.98
**13a**	0.24 ± 0.05	0.27 ± 0.12	0.50 ± 0.12	0.81 ± 0.23	1.12 ± 0.87	1.23 ± 0.92	6.27 ± 2.32
**13b**	0.37 ± 0.13	0.68 ± 0.37	1.03 ± 0.45	1.17 ± 0.74	4.05 ± 0.68	8.66 ± 1.85	44.95 ± 3.91
**13c**	0.26 ± 0.05	0.70 ± 0.12	0.30 ± 0.09	1.40 ± 0.73	27.70 ± 3.96	51.09 ± 3.27	73.46 ± 4.76
**13d**	0.56 ± 0.13	1.50 ± 0.72	3.98 ± 1.03	11.18 ± 2.86	48.65 ± 3.54	65.17 ± 3.91	83.68 ± 4.12
**16a**	0.17 ± 0.04	0.21 ± 0.10	0.35 ± 0.12	0.47 ± 0.11	0.78 ± 0.17	1.23 ± 0.92	3.55 ± 1.03
**16b**	0.19 ± 0.03	0.32 ± 0.26	0.45 ± 0.27	0.51 ± 0.76	0.80 ± 0.65	1.54 ± 0.92	4.37 ± 2.19
**16c**	0.23 ± 0.09	0.44 ± 0.17	0.49 ± 0.21	0.58 ± 0.21	0.82 ± 0.27	1.63 ± 0.98	5.45 ± 1.72
**16d**	0.28 ± 0.06	0.57 ± 0.22	0.73 ± 0.27	0.86 ± 0.51	1.53 ± 0.87	5.94 ± 2.13	21.39 ± 3.12

### SEM images and TEM images of *E. coli* and *S. aureus* morphologies

**Figure 2.**
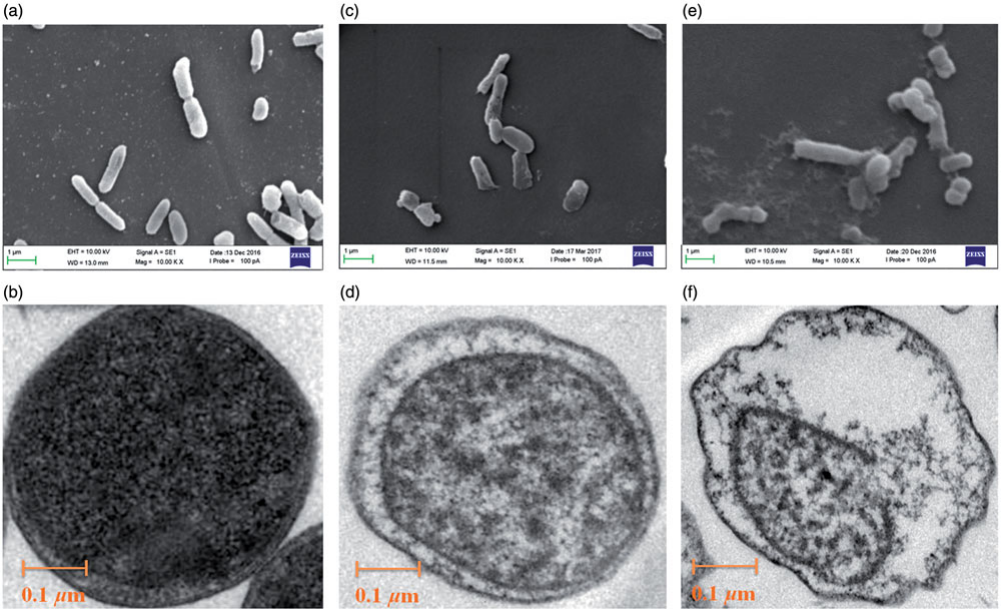
Images of *E. coli* obtained by SEM (a, c & e; scale bar = 1.0 μm) and TEM (b, d & f; scale bar = 0.1 μm) following treatment with culture medium MHB (control; a & b), compound **7d** under the concentration 100 μg/mL for 4 h (c & d), 12 h (e & f).

**Figure 3.**
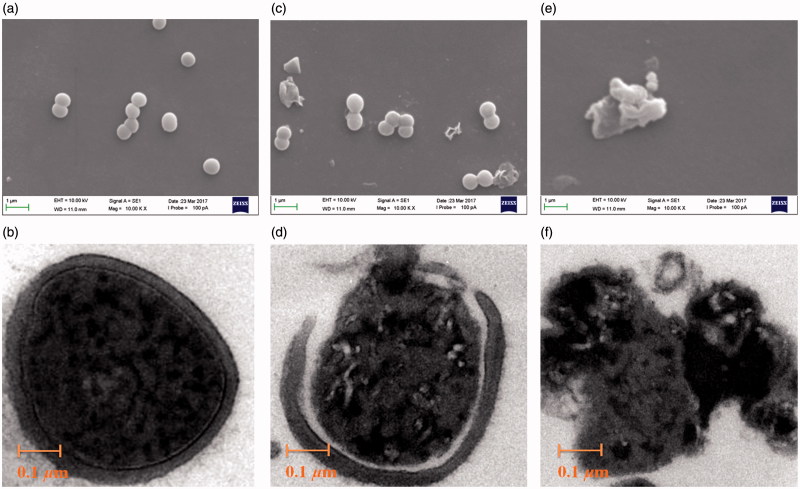
Images of *S. aureus* obtained by SEM (a, c & e; scale bar = 1.0 μm) and TEM (b, d & f; scale bar = 0.1 μm) following treatment with culture medium MHB (control; a & b), compound **7d** under the concentration 100 μg/mL for 4 h (c & d), 12 h (e & f).

Treatment of bacteria with **7d** caused physical and morphological alterations as well as cell wall surface deterioration, and very few intact cells were detected in samples of *E. coli* and *S. aureus*. Scanning electron microscope (SEM) was carried out to follow the morphological alterations of *E. coli* and *S. aureus* and the results are shown in Figures 2 and 3. The *E. coli* were uniformly rod-shaped in their morphology, they had typical dimensions and a smooth cell surface and were present in large numbers (Figure 2(a)). After contacting the addition of compound **7d** (Figures 2(c) and 3(c)) for 4 h, bacteria reveal detrimental effect on the morphology of the cell membrane, showing a large surface collapse and wrinkled abnormalities on the morphology of *E. coli* and *S. aureus*. More obviously, bacterial cytoplasm release from cell membranes and bacteria have been on the verge of death. This may be caused by the antibacterial agents fixing on membrane surfaces^34^. As shown in Figure 2(e) and 3(e), all bacteria died after contacting compound **7d** for 12 h. The alteration of *E. coli* and *S. aureus* morphology may be attributed to the puncturing of cell membranes to result in the release of bacterial cytoplasm^35^. Very few intact cells were detected in samples of *E. coli* and *S. aureus* treated with **7d**.

TEM analysis showed normal *E. coli* and *S. aureus* cell wall and cytoplasmic membrane were intact, and their cytoplasm was homogenously electron dense (Figure 2(b) and 3(b)). A lot of incomplete cells were detected in samples of *E. coli* and *S. aureus* treated with **7d** (Figures 2(d,f) and 3(d,f)). Leakage of intracellular substance was observed in those cells that could be seen. Cells examined by TEM showed regional to complete loss of electron density of both their cell envelope and cytoplasm. In addition, large quantities of electron-dense extracellular material were observed^36^.

## Conclusions

In this study, 24 *N*,*N*-bis(2-hydroxyethyl)-substituted quaternary ammonium salts bromides (**5a-d, 6a-d, 7a-d, 10a-d, 13a-d, 16a-d**) were prepared based on 5-phenyl-1,3,4-oxadiazole-2-thiol (POT) analogues, benzo[*d*]oxazole-2-thiol, benzo[*d*]thiazole-2-thiol, 5-methyl-1,3,4-thiadiazole-2-thiol and *N*,*N*-bis(2-hydroxyethyl) as well as several α,ω-dibromoalkanes. The results displayed that the antibacterial activities and antifungal activities of QASs were basically related to the introduction of POT, chain length and *N*,*N*-bis(2-hydroxyethyl) quaternary ammonium salts. The long-chain and two flexible hydroxyethyl groups in QAS could help QAS effectively pass through cell membrane into the cell to passivate enzyme and damage bacteria. Especially, compounds **5d**, **6d**, **7d**, **10d**, **13d**, **16d** showed unsurpassed antimicrobial activities against all the experimental bacteria and fungi. Meanwhile, introduction of POT fragment in QAS could effectively reduce cytotoxicity and improve antimicrobial activity. Haemolysis assay showed all QASs did not cause haemolysis under the concentration of 50 μg/mL, in which concentration all QASs were safe to be used as antibacterial and antifungal agents. SEM images and TEM images of *E. coli* and *S. aureus* morphologies of **7d** showed antibacterial mechanism might be the antibacterial agents fixing on membrane surfaces and puncturing of cell membranes to result in the release of bacterial cytoplasm. This study provided a facile strategy to design and develop new types of antibacterial and antifungal agents for application as daily or clinical disinfectants, as well as agricultural fungicides.

## Supplementary Material

IENZ_1396456_Supplementary_Material.pdf
