# Supplementary material for: Synthesis, physiochemical property and antimicrobial activity of novel quaternary ammonium salts
Source: J Enzyme Inhib Med Chem. 2017 Nov 17;33(1):98–105. doi: 10.1080/14756366.2017.1396456 (PMC6010013; doi:10.1080/14756366.2017.1396456)
Supplement: IENZ_1396456_Supplementary_Material.pdf [file IENZ_A_1396456_SM7772.pdf]

Synthesis, physiochemical property and antimicrobial activity of novel quaternary ammonium salts

Xianrui Xie[a], Wei Cong[a], Feng Zhao, Hongjuan Li, Wenyu Xin, Chunhua Wang*, Guige Hou*

School of Pharmacy, the Key Laboratory of Prescription Effect and Clinical Evaluation of State Administration of Traditional Chinese Medicine of China, Binzhou Medical University, Yantai, 264003, P. R. China.

******Corresponding author. Tel: +86-535-6913406; fax: +86-535-6913718;*

E-mail address: chunhuawang508@126.com (C.-H. Wang), guigehou@163.com (G.-G. Hou),

[a]*X.-R. Xie and W. Cong contributed equally to this work.*

Experimental

Chemicals and Materials

5-(4-fluorophenyl)-1,3,4-oxadiazole-2-thiol (POTF), 5-(4-trifluoromethylphenyl) -1,3,4-oxadiazole-2-thiol (POTT), 5-(4-*tert*-butylphenyl)-1,3,4-oxadiazole-2-thiol (POTB) were prepared according to a literature[1]. 2-Mercaptobenzoxazole, 2-mercaptobenzothiazole, 2-mercapto-5-methyl-1,3,4-thiadiazole, *N*-methyldiethanolamine (MDEA), *N,N*-dimethylformamide (DMF), 1,6-dibromohexane, 1,8-dibromoctane, 1,10-dibromodecane, 1,12-dibromododecane, benzalkonium bromide (BZK), and chlorhexidine acetate (CA) were purchased from Sinopharm Chemical Reagent Co Ltd and were used as obtained without further purification. *S. Aureus* (ATCC6538), *α-Hemolyticstrep-tococcus* (*α-H-tococcus*, CMCC(B)31005), *β-Hemolyticstrep-tococcus* (CMCC32210), *E. coli* (ATCC25922), *P. aeruginosa* (ATCC9027), *Proteus vulgaris* (CMCC(B)49027) and *Canidia Albicans* (ATCC10231), *Cytospora mandshurica*, *Physalospora piricola*, *Aspergillus niger* (CICC 2041) were purchased from American Type Culture Collection, Manassas, USA. Double-distilled water was used in all the experimental procedures. Infrared (IR) spectra were obtained in the 400-4000 cm^{-1} range using a Perkin-Elmer Frontier Mid-IR FTIR Spectrometer. ^{1}H NMR data were collected using a Bruker Avance 400 spectrometer. All ^{13}C NMR data were collected at 100 MHz using Bruker Avance-400 spectrometer. Chemical shifts are reported in δ relative to TMS. Elemental

analyses were performed on a Perkin-Elmer Model 2400 analyzer. The bacterial morphology were observed by EVO LS15 scanning electron microscopy (SEM, ZEISS, Germany).

Synthesis and characterization of compounds 5a-d

POTF (1.96 g, 10 mmol), 1,6-dibromohexane (12.20 g, 50 mmol) or 1,8-dibromoctane (13.60 g, 50 mmol) or 1,10-dibromodecane (15.00 g, 50 mmol) or 1,12-dibromododecane (16.40 g, 50 mmol) and K_2CO_3 (6.9 g, 50 mmol) were mixture in 100 mL acetonitrile. The mixture was stirred for 4 h at room temperature (monitored by TLC). After filtrated, the filtrate was concentrated and the residue was purified on silica gel by column using petroleum ether/EtOAc (4:1, v/v) as the eluent to afford intermediate **4**. And excess dibromine alkyl was recycled. Intermediate and MDEA (1.19 g, 10 mmol) were mixture in 15 mL DMF. The mixture was stirred for 6 h at 60ºC (monitored by TLC). After added into 200 mL diethyl ether, the mixture was kept at -20ºC overnight, and then filtrated. The residue was recrystallized from methanol and diethyl ether to afford the colorless crystals **5a-d**.

6-((5-(4-fluorophenyl)-1,3,4-oxadiazol-2-yl)thio)-*N*,*N*-bis(2-hydroxyethyl)-*N*-methylhexan-1-aminium bromide (5a)

Yield: 71%. M.p. 74-76℃. IR(cm^{-1}): 3307(br), 3050(s), 1608(s), 1503(s), 1477(s), 1335(s), 1293(s), 1229(s), 1192(s), 1163(s), 1054(s), 1005(w), 961(w), 936(w), 901(w), 861(s), 788(w), 624(s), 602(w), 528(s). ^1H NMR (400 MHz, DMSO-d^6) δ 8.16 - 7.22 (m, 4H, C_6H_5), 5.31 (s, 2H, -OH), 3.80 (t, J = 11.2 Hz, 4H, O-CH_2), 3.45 (s, 4H, N-CH_2), 3.38 (m, 2H, N-CH_2), 3.31 (d, J = 6.3 Hz, 2H, S-CH_2), 3.06 (s, 3H, -CH_3), 1.79 (dt, J = 14.7, 7.3 Hz, 2H, -CH_2), 1.70 (s, 2H, -CH_2), 1.46 (m, 2H, -CH_2), 1.31 (m, 2H, -CH_2). ^{13}C NMR (100 MHz, DMSO-d^6) δ 164.73, 164.47(d, $1J_{CF}$ = 249 Hz), 164.36, 129.51(d, $3J_{CF}$ = 9.2 Hz), 120.17(d, $4J_{CF}$ = 3.1 Hz), 117.13(d, $2J_{CF}$ = 22.5 Hz), 63.58, 62.68, 55.17, 49.42, 32.29, 29.06, 27.68, 25.57, 21.83. Elemental analysis (%) calcd. for $C_{19}H_{29}BrFN_3O_3S$ (478.42): C 47.70, H 6.11, N 8.78, S 6.70; Found: C 47.66, H 6.08, N 8.72, S 6.64.

8-((5-(4-fluorophenyl)-1,3,4-oxadiazol-2-yl)thio)-*N*,*N*-bis(2-hydroxyethyl)-*N*-methyloctan-1-aminium bromide (5b)

Yield: 67%. M.p. 81-82℃. IR(cm^{-1}): 3294(br), 2931(m), 2857(m), 1610(s), 1501(s), 1480(s), 1222(s), 1189(m), 1161(m), 1099(w), 1069(s), 1000(w), 955(w), 864(s), 731(s), 696(m), 622(s), 526(m). ^1H NMR (400 MHz, DMSO-d^6) δ 8.17 - 7.29 (m, 4H, C_6H_5), 5.24 (s, 2H,

-OH), 3.81 (d, J = 3.0 Hz, 4H, O-CH_2), 3.42 (s, 2H, S-CH_2), 3.33 (m, 6H, N-CH_2), 3.07 (s, 3H, -CH_3), 1.76 (dd, J = 14.1, 7.1 Hz, 2H, -CH_2), 1.67 (s, 2H, -CH_2), 1.41 (m, 2H, -CH_2), 1.35 - 1.23 (m, 6H, -CH_2). ^{13}C NMR (100 MHz, DMSO-d^6) δ 164.73, 164.48(d, $1J_{CF}$ = 249 Hz), 164.38, 129.51(d, $3J_{CF}$ = 9.2 Hz), 120.18(d, $4J_{CF}$ = 3.2 Hz), 117.14(d, $2J_{CF}$ = 22.5 Hz), 63.56, 62.78, 55.17, 49.39, 32.44, 29.30, 28.73, 28.54, 28.11, 26.10, 21.94. Elemental analysis (%) calcd. for $C_{21}H_{33}BrFN_3O_3S$ (506.47): C 49.80, H 6.57, N 8.30, S 6.33; Found: C 49.76, H 6.48, N 8.22, S 6.24.

10-((5-(4-fluorophenyl)-1,3,4-oxadiazol-2-yl)thio)-*N,N*-bis(2-hydroxyethyl)-*N*-methyldecan-1-aminium bromide (5c)

Yield: 61%. M.p. 102-103℃. IR(cm^{-1}): 3314(br), 2928(m), 2855(m), 1610(s), 1504(s), 1471(s), 1363(w), 1227(s), 1202(w), 1183(w), 1161(m), 1070(s), 1053(m), 923(w), 854(s), 731(m), 696(w), 624(m), 523(w). ^{1}H NMR (400 MHz, DMSO-d^6) δ 8.16 - 7.29 (m, 4H, C_6H_5), 5.25 (s, 2H, -OH), 3.87 (t, J = 37.0 Hz, 4H, O-CH_2), 3.45 (m, 6H, N-CH_2), 3.29 (s, 2H, S-CH_2), 3.07(s, 3H, -CH_3), 1.76 (m, 2H, -CH_2), 1.66 (s, 2H, -CH_2), 1.40 (s, 2H, -CH_2), 1.27 (s, 10H, -CH_2). ^{13}C NMR (100 MHz, DMSO-d^6) δ 164.72, 164.48(d, $1J_{CF}$ = 249 Hz), 164.39, 129.51 (d, $3J_{CF}$ = 9.2 Hz), 120.18 (d, $4J_{CF}$ = 3.2 Hz), 117.13 (d, $2J_{CF}$ = 22.5 Hz), 63.58, 62.80, 55.18, 49.39, 32.47, 29.37, 29.16, 29.12, 28.88, 28.77, 28.22, 26.18, 21.98. Elemental analysis (%) calcd. for $C_{23}H_{37}BrFN_3O_3S$ (534.53): C 51.68, H 6.98, N 7.86, S 6.00; Found: C 51.62, H 6.88, N 7.68, S 6.04.

12-((5-(4-fluorophenyl)-1,3,4-oxadiazol-2-yl)thio)-*N,N*-bis(2-hydroxyethyl)-*N*-methyldodecan-1-aminium bromide (5d)

Yield: 60%. M.p. 98-100℃. IR(cm^{-1}): 3309(br), 2921(m), 2854(m), 1610(s), 1503(s), 1470(s), 1369(w), 1227(s), 1184(s), 1161(w), 1085(w), 1068(m), 1053(m), 991(s), 915(w), 853(s), 730(m), 696(w), 623(m), 522(w). ^{1}H NMR (400 MHz, DMSO-d^6) δ 8.03 - 7.45 (m, 4H, C_6H_5), 5.24 (s, 2H, -OH), 3.80 (t, J = 10.9 Hz, 4H, O-CH_2), 3.44 (s, 4H, N-CH_2), 3.36 (m, 2H, N-CH_2), 3.29 (s, 2H, S-CH_2), 3.07 (s, 3H, -CH_3), 1.74 (dd, J = 14.1, 7.1 Hz, 2H, -CH_2), 1.66 (s, 2H, -CH_2), 1.40 (s, 2H, -CH_2), 1.24 (s, 14H, -CH_2). ^{13}C NMR (100 MHz, DMSO-d^6) δ 164.71, 164.47(d, $1J_{CF}$ = 249 Hz), 164.38, 129.50 (d, $3J_{CF}$ = 9.2 Hz), 120.19 (d, $4J_{CF}$ = 3.1 Hz), 117.12(d, $2J_{CF}$ = 22.5 Hz), 63.56, 62.79, 55.17, 49.39, 32.47, 29.36, 29.33, 29.29,

29.23, 28.93, 28.80, 28.21, 26.20, 21.99. Elemental analysis (%) calcd. for $C_{25}H_{41}BrFN_3O_3S$ (562.58): C 53.37, H 7.35, N 7.47, S 5.70; Found: C 53.52, H 7.58, N 7.28, S 5.74.

Synthesis and characterization of compounds 6a-d

POTT (2.46 g, 10 mmol), or 1,6-dibromohexane (12.20 g, 50 mmol) or 1,8-dibromoctane (13.60 g, 50 mmol) or 1,10-dibromodecane (15.00 g, 50 mmol) or 1,12-dibromododecane (16.40 g, 50 mmol) and K_2CO_3 (6.9 g, 50 mmol) were mixture in 100 mL acetonitrile. The mixture was stirred for 4 h at room temperature (monitored by TLC). After filtrated, the filtrate was concentrated and the residue was purified on silica gel by column using petroleum ether/EtOAc (4:1, v/v) as the eluent to afford intermediate **4**. And excess dibromine alkyl was recycled. Intermediate and MDEA (1.19 g, 10 mmol) were mixture in 15 mL DMF. The mixture was stirred for 6 h at 60ºC (monitored by TLC). After added into 200 mL diethyl ether, the mixture was kept at -20ºC overnight, and then filtrated. The residue was recrystallized from methanol and diethyl ether to afford the colorless crystals **6a-d**.

***N,N*-bis(2-hydroxyethyl)-*N*-methyl-6-((5-(4-(trifluoromethyl)phenyl)-1,3,4-oxadiazol-2-yl)thio)hexan-1-aminium bromide (6a)**

Yield: 66%. M.p. 93-94℃. IR(cm^{-1}): 3239(br), 1559(w), 1470(s), 1415(m), 1324(s), 1193(m), 1163(s), 1112(s), 1077(s), 1051(s), 1004(w), 947(m), 927(m), 884(m), 845(s), 833(m), 794(w), 780(w), 731(s), 702(s), 645(m), 593(m). ^1H NMR (400 MHz, DMSO-d^6) δ 8.18-7.97 (m, 4H, C_6H_5), 5.32 (s, 2H, -OH), 3.79 (m, J = 13.7 Hz, 4H, O-CH_2), 3.39 (m, 6H, N-CH_2), 3.33 (s, 2H, S-CH_2), 3.08 (s, 3H, -CH_3), 1.81 (m, 2H, -CH_2), 1.70 (s, 2H, -CH_2), 1.47 (m, 2H, -CH_2), 1.31 (m, 2H, -CH_2). ^{13}C NMR (100 MHz, DMSO-d^6) δ 165.31, 164.45, 131.87 (d, $1J_{CF}$ = 32 Hz), 127.64, 127.24, 126.83 (d, $2J_{CF}$ = 4 Hz), 125.49, 122.78, 63.61, 62.71, 55.18, 49.42, 32.32, 29.03, 27.69, 25.57, 21.83. Elemental analysis (%) calcd. for $C_{20}H_{29}BrF_3N_3O_3S$ (528.43): C 45.46, H 5.53, N 7.95, S 6.07; Found: C 45.52, H 5.58, N 7.82, S 6.34.

***N,N*-bis(2-hydroxyethyl)-*N*-methyl-8-((5-(4-(trifluoromethyl)phenyl)-1,3,4-oxadiazol-2-yl)thio)octan-1-aminium bromide (6b)**

Yield: 60%. M.p. 78-80℃. IR(cm^{-1}): 3325(br), 2933(w), 2856(w), 1563(w), 1468(s), 1416(w), 1319(s), 1177(s), 1135(s), 1110(m), 1074(s), 1017(m), 966(m), 900(m), 845(s), 793(w), 779(w), 747(w), 703(s), 596(m). ^1H NMR (400 MHz, DMSO-d^6) δ 8.18-7.97 (m, 4H, C_6H_5), 5.25 (s, 2H, -OH), 3.81 (d, J = 4.0 Hz, 4H, O-CH_2), 3.43 (s, 2H, S-CH_2), 3.40 (m, 6H,

N-CH_2), 3.02 (s, 3H, -CH_3), 1.78(m, 2H, -CH_2), 1.67 (s, 2H, -CH_2), 1.42 (s, 2H, -CH_2), 1.32 (m, 4H, -CH_2), 1.26 (d, J = 5.7 Hz, 2H, -CH_2). ^{13}C NMR (100 MHz, DMSO-d^6) δ 165.33, 164.44, 131.87 (d, $1J_{CF}$ = 32 Hz), 127.64, 127.25, 126.83 (d, $2J_{CF}$ = 4 Hz), 125.49, 122.78, 63.57, 62.79, 55.18, 49.39, 32.47, 29.26, 28.73, 28.55, 28.13, 26.10, 21.95. Elemental analysis (%) calcd. for $C_{22}H_{33}BrF_3N_3O_3S$ (556.48): C 47.48, H 5.98, N 7.55, S 5.76; Found: C 47.32, H 5.86, N 7.32, S 5.82.

***N,N*-bis(2-hydroxyethyl)-*N*-methyl-10-((5-(4-(trifluoromethyl)phenyl)-1,3,4-oxadiazol-2-yl)thio)decan-1-aminium bromide (6c)**

Yield: 58%. M.p. 102-103℃. IR(cm^{-1}): 3252(br), 2922(w), 2848(w), 1559(w), 1467(s), 1416(w), 1320(s), 1165(m), 1143(s), 1113(m), 1072(s), 1009(m), 974(w), 960(w), 940(m), 851(s), 779(m), 747(m), 721(m), 705(s), 639(m), 596(m). ^{1}H NMR (400 MHz, DMSO-d^6) δ 8.17 - 7.96 (m, 4H, C_6H_5), 5.25 (s, 2H, -OH), 3.81 (d, J = 3.4 Hz, 4H, O-CH_2), 3.39 (m, 6H, N-CH_2), 3.32 (s, 2H, S-CH_2), 3.07 (s, 3H, -CH_3), 1.75 (dt, J = 15.9, 7.8 Hz, 2H, -CH_2), 1.66 (s, 2H, -CH_2), 1.41 (s, 2H, -CH_2), 1.27 (s, 10H, -CH_2). ^{13}C NMR (100 MHz, DMSO-d^6) 165.34, 164.42, 131.86 (d, $1J_{CF}$ = 32 Hz), 127.63, 127.23, 126.82 (d, $2J_{CF}$ = 4 Hz), 125.49, 122.78, 63.56, 62.80, 55.17, 49.40, 32.48, 29.34, 29.18, 29.14, 28.89, 28.78, 28.25, 26.18, 21.99. Elemental analysis (%) calcd. for $C_{24}H_{37}BrF_3N_3O_3S$ (584.53): C 49.31, H 6.38, N 7.19, S 5.49; Found: C 49.51, H 6.56, N 7.35, S 5.32.

***N,N*-bis(2-hydroxyethyl)-*N*-methyl-12-((5-(4-(trifluoromethyl)phenyl)-1,3,4-oxadiazol-2-yl)thio)dodecan-1-aminium bromide (6d)**

Yield: 56%. M.p. 88-90℃. IR(cm^{-1}): 3257(br), 2923(w), 2848(w), 1467(s), 1416(w), 1321(s), 1181(w), 1166(m), 1145(s), 1113(m), 1072(s), 1009(m), 959(w), 854(s), 747(m), 723(w), 706(s), 638(w), 598(w). ^{1}H NMR (400 MHz, DMSO-d^6) δ 8.18 - 7.97 (m, 4H, C_6H_5), 5.25 (s, 2H, -OH), 3.79 (t, J = 12.4 Hz, 4H, O-CH_2), 3.44 (s, 2H, S-CH_2), 3.39 (m, 6H, N-CH_2), 3.07 (s, 3H, -CH_3), 1.77 (m, 2H, -CH_2), 1.66 (s, 2H, -CH_2), 1.40 (s, 2H, -CH_2), 1.24 (s, 14H, -CH_2). ^{13}C NMR (100 MHz, DMSO-d^6) δ 165.33, 164.42, 131.86 (d, $1J_{CF}$ = 32 Hz), 127.63, 127.24, 126.82 (d, $2J_{CF}$ = 4 Hz), 125.49, 122.78, 63.57, 62.81, 55.17, 49.40, 32.49, 29.34, 29.30, 29.23, 28.93, 28.81, 28.23, 26.20, 21.99. Elemental analysis (%) calcd. for $C_{26}H_{41}BrF_3N_3O_3S$ (612.59): C 50.98, H 6.75, N 6.86, S 5.23; Found: C 50.82, H 6.86, N 6.55, S 5.36.

Synthesis and characterization of compounds 7a-d

POTB (2.34 g, 10 mmol), 1,6-dibromohexane (12.20 g, 50 mmol) or 1,8-dibromoctane (13.60 g, 50 mmol) or 1,10-dibromodecane (15.00 g, 50 mmol) or 1,12-dibromododecane (16.40 g, 50 mmol) and K_2CO_3 (6.9 g, 50 mmol) were mixture in 100 mL acetonitrile. The mixture was stirred for 4 h at room temperature (monitored by TLC). After filtrated, the filtrate was concentrated and the residue was purified on silica gel by column using petroleum ether/EtOAc (4:1, v/v) as the eluent to afford intermediate **4**. And excess dibromine alkyl was recycled. Intermediate and MDEA (1.19 g, 10 mmol) were mixture in 15 mL DMF. The mixture was stirred for 6 h at 60ºC (monitored by TLC). After added into 200 mL diethyl ether, the mixture we kept at -20ºC overnight, and then filtrated. The residue was recrystallized from methanol and diethyl ether to afford the colorless crystals **7a-d**.

6-((5-(4-(*tert*-butyl)phenyl)-1,3,4-oxadiazol-2-yl)thio)-*N*,*N*-bis(2-hydroxyethyl)-*N*-methyl hexan-1-aminium bromide (7a)

Yield: 64%. M.p. 78-80℃. IR(cm^{-1}): 3305(br), 2953(w), 2865(w), 1615(m), 1585(w), 1473(s), 1364(m), 1267(m), 1199(w), 1182(s), 1110(m), 1066(m), 954(s), 847(s), 749(m), 739(m), 709(s), 560(m). ^{1}H NMR (400 MHz, DMSO-d^6) δ 7.88 - 7.61 (m, 4H, C_6H_5), 5.24 (s, 2H, -OH), 3.82 (m, 4H, O-CH_2), 3.45 (s, 4H, N-CH_2), 3.37 (m, 2H, N-CH_2), 3.32 (d, 2H, S-CH_2), 3.09 (s, 3H, N-CH_3), 1.78 (dd, J = 13.8, 7.0 Hz, 2H, -CH_2), 1.71 (m, 2H, -CH_2), 1.46 (d, J = 5.9 Hz, 2H, -CH_2), 1.31 (s, 2H, -CH_2), 1.31 (s, 9H, -CH_3). ^{13}C NMR (100 MHz, DMSO-d^6) δ 165.47, 164.02, 155.32, 126.69, 126.63, 120.75, 63.58, 62.69, 55.17, 49.42, 35.25, 32.29, 31.21, 29.08, 27.69, 25.57, 21.83. Elemental analysis (%) calcd. for $C_{23}H_{38}BrN_3O_3S$ (516.54): C 53.48, H 7.42, N 8.13, S 6.21; Found: C 53.52, H 7.36, N 8.25, S 6.35.

8-((5-(4-(*tert*-butyl)phenyl)-1,3,4-oxadiazol-2-yl)thio)-*N*,*N*-bis(2-hydroxyethyl)-*N*-methyl octan-1-aminium bromide (7b)

Yield: 57%. M.p. 71-72℃. IR(cm^{-1}): 3317(br), 2929(w), 2859(w), 1616(w), 1585(w), 1466(s), 1364(m), 1322(m), 1266(m), 1179(s), 1108(m), 1092(m), 1064(s), 988(m), 954(m), 900(m), 841(s), 747(m), 738(w), 706(s), 554(m). ^{1}H NMR (400 MHz, DMSO-d^6) δ 7.86 -7.60 (m, 4H, C_6H_5), 5.44 (s, 2H, -OH), 3.81 (m, 4H, O-CH_2), 3.45 (d, 4H, N-CH_2), 3.37 (m, 2H, N-CH_2), 3.30 (t, J = 7.1 Hz, 2H, S-CH_2), 3.09 (s, 3H, N-CH_3), 1.76 (dt, J = 13.7, 6.7 Hz,

2H, -CH_2), 1.68 (s, 2H, -CH_2), 1.41 (s, 2H, -CH_2), 1.24 (s, 6H, -CH_2), 1.24 (s, 9H, -CH_3). ^{13}C NMR (100 MHz, DMSO-*d*^{6}) δ 165.45, 164.03, 155.31, 126.68, 126.62, 120.76, 63.54, 62.77, 55.13, 49.41, 35.24, 32.44, 31.21, 29.31, 28.74, 28.57, 28.13, 26.11, 21.94. Elemental analysis (%) calcd. for $C_{25}H_{42}BrN_3O_3S$ (544.59): C 55.14, H 7.77, N 7.72, S 5.89; Found: C 55.33, H 7.56, N 7.65, S 5.75.

10-((5-(4-(*tert*-butyl)phenyl)-1,3,4-oxadiazol-2-yl)thio)-*N*,*N*-bis(2-hydroxyethyl)-*N*-methyldecan-1-aminium bromide (7c)

Yield: 55%. M.p. 78-80℃. IR(cm^{-1}): 3307(br), 2925(m), 2855(w), 1663(w), 1616(w), 1467(s), 1366(w), 1268(w), 1182(s), 1109(w), 1066(s), 997(m), 953(m), 841(s), 739(m), 707(s), 555(m). ^{1}H NMR (400 MHz, DMSO-*d*^{6}) δ 7.88 - 7.60 (m, 4H, C_6H_5), 5.24 (s, 2H, -OH), 3.82 (m, 4H, O-CH_2), 3.44 (d, 4H, N-CH_2), 3.32 (m, 2H, N-CH_2), 3.28 (d, *J* = 6.5 Hz, 2H, S-CH_2), 3.08 (s, 3H, N-CH_3), 1.75 (d, *J* = 6.5 Hz, 2H, -CH_2), 1.67 (s, 2H, -CH_2), 1.40 (s, 2H, -CH_2), 1.31 (s, 10H, -CH_2), 1.27 (s, 9H, -CH_3). ^{13}C NMR (100 MHz, DMSO-*d*^{6}) δ 165.47, 164.03, 155.33, 126.67, 126.63, 120.78, 63.60, 62.85, 55.19, 49.42, 35.24, 32.50, 31.22, 29.39, 29.16, 29.12, 28.86, 28.77, 28.24, 26.19, 22.00. Elemental analysis (%) calcd. for $C_{27}H_{46}BrN_3O_3S$ (572.64): C 56.63, H 8.10, N 7.34, S 5.60; Found: C 56.33, H 8.26, N 7.55, S 5.75.

12-((5-(4-(*tert*-butyl)phenyl)-1,3,4-oxadiazol-2-yl)thio)-*N*,*N*-bis(2-hydroxyethyl)-*N*-methyldodecan-1-aminium bromide (7d)

Yield: 51%. M.p. 82-84℃. IR(cm^{-1}): 3297(br), 2925(m), 2854(w), 1617(w), 1468(s), 1366(w), 1268(w), 1182(s), 1109(w), 1065(s), 1017(w), 996(m), 953(m), 842(s), 739(m), 724(w), 702(s), 600(w), 555(m). ^{1}H NMR (400 MHz, DMSO-*d*^{6}) δ 7.88 - 7.60 (m, 4H, C_6H_5), 5.25 (s, 2H, -OH), 3.81 (s, 4H, O-CH_2), 3.44 (m, 2H, N-CH_2), 3.35 (d, 4H, N-CH_2), 3.29 (t, *J* = 7.1 Hz, 2H, S-CH_2), 3.07 (s, 3H, N-CH_3), 1.75 (dt, *J* = 14.4, 7.2 Hz, 2H, -CH_2), 1.66 (s, 2H, -CH_2), 1.39 (s, 2H, -CH_2), 1.31 (s, 9H, -CH_3), 1.24 (s, 14H, -CH_2). ^{13}C NMR (100 MHz, DMSO-*d*^{6}) δ 165.45, 164.03, 155.32, 126.67, 126.62, 120.77, 63.57, 62.82, 55.18, 49.40, 35.24, 32.47, 31.21, 29.40, 29.33, 29.29, 29.23, 28.92, 28.81, 28.23, 26.21, 22.00. Elemental

analysis (%) calcd. for $C_{29}H_{50}BrN_3O_3S$ (600.69): C 57.98, H 8.39, N 7.00, S 5.34; Found: C 57.86, H 8.56, N 7.15, S 5.65.

Synthesis and characterization of compounds 10a-d

2-Mercaptobenzoxazole (1.52 g, 10 mmol), 1,6-dibromohexane (12.20 g, 50 mmol) or 1,8-dibromoctane (13.60 g, 50 mmol) or 1,10-dibromodecane (15.00 g, 50 mmol) or 1,12-dibromododecane (16.40 g, 50 mmol) and K_2CO_3 (6.9 g, 50 mmol) were mixture in 100 mL acetonitrile. The mixture was stirred for 3 h at room temperature (monitored by TLC). After filtrated, the filtrate was concentrated and the residue was purified on silica gel by column using petroleum ether/EtOAc (8:1, v/v) as the eluent to afford intermediate **9**. And excess dibromine alkyl was recycled. Intermediate and MDEA (1.19 g, 10 mmol) were mixture in 15 mL DMF. The mixture was stirred for 6 h at 50ºC (monitored by TLC). After added into 200 mL diethyl ether, the mixture was kept at -20ºC overnight, and then filtrated. The residue was recrystallized from methanol and diethyl ether to afford the colorless crystals **10a-d**.

6-(benzo[*d*]oxazol-2-ylthio)-*N,N*-bis(2-hydroxyethyl)-*N*-methylhexan-1-aminium bromide (10a)

Yield: 65%. M.p. 95-96℃. IR(cm^{-1}): 3275(br), 3021(s), 2940(m), 2856(m), 1501(s), 1469(s), 1237(s), 1219(s), 1134(s), 1101(s), 1083(s), 1046(s), 1006(m), 965(s), 929(s), 807(s), 763(s), 756(s), 734(s). ^{1}H NMR (400 MHz, DMSO-d^6) δ 7.67 - 7.28 (m, 4H, C_6H_5), 5.24 (t, J = 4.9 Hz, 2H, -OH), 3.82 (d, J = 3.4 Hz, 4H, O-CH_2), 3.45 (s, 4H, N-CH_2), 3.38 (dd, J = 9.7, 3.3 Hz, 2H, N-CH_2), 3.33 (d, J = 2.6 Hz, 2H, S-CH_2), 3.08 (s, 3H, N-CH_3), 1.84 - 1.77 (m, 2H, -CH_2), 1.70 (s, 2H, -CH_2), 1.51 - 1.44 (m, 2H, -CH_2), 1.32 (d, J = 6.9 Hz, 2H, -CH_2). ^{13}C NMR (100 MHz, DMSO-d^6) δ 164.87, 151.62, 141.72, 125.00, 124.61, 118.60, 110.57, 63.62, 62.73, 55.18, 49.43, 31.98, 29.01, 27.78, 25.63, 21.86. Elemental analysis (%) calcd. for $C_{20}H_{33}BrN_2O_3S$ (459.92): C 52.06, H 7.21, N 6.07, S 6.95; Found: C 52.12, H 7.31, N 6.02, S 6.33.

8-(benzo[*d*]oxazol-2-ylthio)-*N,N*-bis(2-hydroxyethyl)-*N*-methyloctan-1-aminium bromide (10b)

Yield: 71%. M.p. 125-127℃. IR(cm^{-1}): 3353(br), 3262(m), 2937(m), 2857(s), 1507(s), 1461(s), 1427(s), 1239(s), 1210(s), 1142(s), 1102(s), 1051(s), 1034(s), 948(s), 925(s), 806(s),

771(s), 726(s), 623(s), 589(m). ^{1}H NMR (400 MHz, DMSO-*d*^{6}) δ 7.64 - 7.32 (m, 4H, C_6H_5), 5.27 (s, 2H, -OH), 3.82 (d, *J* = 3.8 Hz, 4H, O-CH_2), 3.45 (m, *J* = 2.2 Hz, 4H, N-CH_2), 3.38(m, 2H, N-CH_2), 3.32(t, 2H, S-CH_2), 3.08 (s, 3H, N-CH_3), 1.83 - 1.73 (m, 2H, -CH_2), 1.68 (s, 2H, -CH_2), 1.42 (s, 2H, -CH_2), 1.32 (s, 4H, -CH_2), 1.26 (d, *J* = 6.4 Hz, 2H, -CH_2). ^{13}C NMR (100 MHz, DMSO-*d*^{6}) δ 164.52, 151.20, 141.31, 124.62, 124.22, 118.20, 110.18, 63.15, 62.39, 54.76, 49.01, 31.71, 28.84, 28.36, 28.22, 27.82, 25.72, 21.56. Elemental analysis (%) calcd. for $C_{24}H_{41}BrN_2O_3S$ (515.92): C 55.70, H 7.98, N 5.41, S 6.20; Found: C 55.68, H 7.88, N 5.32, S 6.26.

10-(benzo[*d*]oxazol-2-ylthio)-*N,N*-bis(2-hydroxyethyl)-*N*-methyldecan-1-aminium bromide (10c)

Yield: 63%. M.p. 128-129℃. IR(cm^{-1}): 3359(br), 3267(w), 2933(w), 2856(w), 1505(s), 1490(m), 1457(s), 1424(s), 1230(m), 1211(m), 1142(s), 1101(s), 1054(s), 1035(s), 943(m), 925(s), 874(s), 807(m), 771(s), 730(s). ^{1}H NMR (400 MHz, DMSO-d^{6}) δ 7.63 - 7.28 (m, 4H, C_6H_5), 5.26 (t, *J* = 4.6 Hz, 2H, -OH), 3.81 (d, *J* = 3.8 Hz, 4H, O-CH_2), 3.44 (m, 4H, N-CH_2), 3.36 (m, 2H, N-CH_2), 3.31 (t, 2H, S-CH_2), 3.08 (s, 3H, N-CH_3), 1.82 - 1.72 (m, 2H, -CH_2), 1.66 (s, 2H, -CH_2), 1.40 (d, *J* = 6.2 Hz, 2H, -CH_2), 1.27 (s, 10H, -CH_2). ^{13}C NMR (100 MHz, DMSO-d^{6}) δ 164.90, 151.61, 141.74, 124.99, 124.59, 118.58, 110.54, 63.60, 62.85, 55.18, 49.42, 32.13, 29.27, 29.16, 29.12, 28.87, 28.80, 28.31, 26.19, 22.00. Elemental analysis (%) calcd. for $C_{22}H_{37}BrN_2O_3S$ (487.92): C 53.98, H 7.62, N 5.72, S 6.55; Found: C 54.02, H 7.58, N 5.68, S 6.52.

12-(benzo[*d*]oxazol-2-ylthio)-*N,N*-bis(2-hydroxyethyl)-*N*-methyldodecan-1-aminium bromide (10d)

Yield: 71%. M.p. 125-127℃. IR(cm^{-1}): 3353(br), 3262(m), 2937(m), 2857(s), 1507(s), 1461(s), 1427(s), 1239(s), 1210(s), 1142(s), 1102(s), 1051(s), 1034(s), 948(s), 925(s), 806(s), 771(s), 726(s), 623(s), 589(w). ^{1}H NMR (400 MHz, DMSO-d^{6}) δ 7.63 - 7.32 (m, 4H, C_6H_5), 5.27 (s, 2H, -OH), 3.81 (s, 4H, O-CH_2), 3.44 (d, *J* = 1.9 Hz, 4H, N-CH_2), 3.36 (d, *J* = 11.3 Hz, 2H, N-CH_2), 3.30 (d, *J* = 7.3 Hz, 2H, S-CH_2), 3.08 (s, 3H, N-CH_3), 1.81 - 1.72 (m, 2H, -CH_2), 1.66 (s, 2H, -CH_2), 1.40 (d, *J* = 6.5 Hz, 2H, -CH_2), 1.24 (s, 14H, -CH_2). ^{13}C NMR (100 MHz, DMSO-d^{6}) δ 164.90, 151.61, 141.74, 124.98, 124.58, 118.57, 110.53, 63.60, 62.85, 55.18, 49.41, 32.13, 29.31, 29.26, 29.21, 28.91, 28.82, 28.29, 26.21, 22.01. Elemental

analysis (%) calcd. for $C_{24}H_{41}BrN_2O_3S$ (515.92): C 55.70, H 7.98, N 5.41, S 6.20; Found: C 55.63, H 7.86, N 5.32, S 6.18.

Synthesis and characterization of compounds 13a-d

2-Mercaptobenzothiazole (1.67 g, 10 mmol), 1,6-dibromohexane (12.20 g, 50 mmol) or 1,8-dibromoctane (13.60 g, 50 mmol) or 1,10-dibromodecane (15.00 g, 50 mmol) or 1,12-dibromododecane (16.40 g, 50 mmol) and K_2CO_3 (6.9 g, 50 mmol) were mixture in 100 mL acetonitrile. The mixture was stirred for 3 h at room temperature (monitored by TLC). After filtrated, the filtrate was concentrated and the residue was purified on silica gel by column using petroleum ether/EtOAc (20:1, v/v) as the eluent to afford intermediate **12**. And excess dibromine alkyl was recycled. Intermediate and MDEA (1.19 g, 10 mmol) were mixture in 15 mL DMF. The mixture was stirred for 6 h at 50ºC (monitored by TLC). After added into 200 mL diethyl ether, the mixture was kept at -20ºC overnight, and then filtrated. The residue was recrystallized from methanol and diethyl ether to afford the colorless crystals **13a-d**.

6-(benzo[*d*]thiazol-2-ylthio)-*N,N*-bis(2-hydroxyethyl)-*N*-methylhexan-1-aminium bromide (13a)

Yield: 67%. M.p. 142-143℃. IR(cm^{-1}): 3290(br), 2934(m), 2866(w), 1464(s), 1430(s), 1312(s), 1244(s), 1097(m), 1080(s), 1001(m), 961(s), 922(s), 859(m), 775(s), 741(s), 707(s), 673(s), 611(m). ^1H NMR (400 MHz, DMSO-d^6) δ 8.04 - 7.32 (m, 4H, C_6H_5), 5.24 (t, J = 4.9 Hz, 2H, -OH), 3.81 (d, J = 3.6 Hz, 4H, O-CH_2), 3.44 (d, J = 1.8 Hz, 4H, N-CH_2), 3.38 (d, J = 6.7 Hz, 2H, N-CH_2), 3.34 (d, J = 10.5 Hz, 2H, S-CH_2), 3.08 (s, 3H, N-CH_3), 1.79 (dt, J = 14.5, 7.2 Hz, 2H, -CH_2), 1.70 (s, 2H, -CH_2), 1.52 - 1.43 (m, 2H, -CH_2), 1.36 - 1.27 (m, 2H, -CH_2). ^{13}C NMR (100 MHz, DMSO-d^6) δ 167.18, 153.19, 134.89, 126.79, 124.84, 122.20, 121.49, 63.62, 62.74, 55.19, 49.43, 33.12, 28.93, 27.92, 25.66, 21.88. Elemental analysis (%) calcd. for $C_{18}H_{29}BrN_2O_2S_2$ (447.92): C 48.10, H 6.50, N 6.23, S 14.27; Found: C 48.12, H 6.42, N 6.12, S 14.22.

8-(benzo[*d*]thiazol-2-ylthio)-*N,N*-bis(2-hydroxyethyl)-*N*-methyloctan-1-aminium bromide (13b)

Yield: 67%. M.p. 87-89℃. IR(cm^{-1}): 3307(br), 2928(m), 2862(m), 1456(m), 1434(s), 1310(s), 1244(s), 1072(s), 1050(s), 1025(s),1003(s), 952(s), 915(s), 765(s), 736(s), 703(s), 670(s),

627(m), 594(m). ^{1}H NMR (400 MHz, DMSO-d^6) δ 8.01 - 7.36 (m, 4H, C_6H_5), 5.24 (s, 2H, -OH), 3.82 (s, 4H, O-CH_2), 3.45 (s, 4H, N-CH_2), 3.36 (s, 2H, N-CH_2), 3.36 - 3.30 (m, 2H, S-CH_2), 3.08 (s, 3H, N-CH_3), 1.77 (s, 2H, -CH_2), 1.68 (s, 2H, -CH_2), 1.43 (s, 2H, -CH_2), 1.32 (s, 6H, -CH_2). ^{13}C NMR (100 MHz, DMSO-d^6) δ 167.23, 153.19, 134.88, 126.79, 124.82, 122.21, 121.47, 63.55, 62.78, 55.17, 49.41, 33.24, 29.13, 28.75, 28.62, 28.33, 26.11, 21.96. Elemental analysis (%) calcd. for $C_{20}H_{33}BrN_2O_2S_2$ (475.92): C 50.30, H 6.97, N 5.87, S 13.43; Found: C 50.26, H 6.86, N 5.68, S 13.26.

10-(benzo[*d*]thiazol-2-ylthio)-*N,N*-bis(2-hydroxyethyl)-*N*-methyldecan-1-aminium bromide (13c)

Yield: 65%. M.p. 111-112℃. IR(cm^{-1}): 3282(br), 2935(m), 2858(m), 1492(s), 1456(s), 1427(s), 1368(s), 1310(s), 1222(m), 1098(s), 1054(s), 1032(s), 1007(s), 952(m), 879(s), 780(s), 733(s), 660(s). ^{1}H NMR (400 MHz, DMSO-d^6) δ 8.05 - 7.31 (m, 4H, C_6H_5), 5.24 (t, J = 4.7 Hz, 2H, -OH), 3.81 (d, J = 3.8 Hz, 4H, O-CH_2), 3.43 (d, J = 2.3 Hz, 4H, N-CH_2), 3.36 (d, J = 6.8 Hz, 2H, N-CH_2), 3.33 (d, J = 5.2 Hz, 2H, S-CH_2), 3.07 (s, 3H, N-CH_3), 1.81 - 1.71 (m, 2H, -CH_2), 1.66 (s, 2H, -CH_2), 1.42 (d, J = 6.9 Hz, 2H, -CH_2), 1.27 (s, 10H, -CH_2). ^{13}C NMR (100 MHz, DMSO-d^6) δ 167.24, 153.18, 134.88, 126.78, 124.82, 122.21, 121.46, 63.55, 62.78, 55.17, 49.39, 33.24, 29.17, 29.14, 28.89, 28.83, 28.43, 26.19, 21.99. Elemental analysis (%) calcd. for $C_{22}H_{37}BrN_2O_2S_2$ (503.92): C 52.26, H 7.38, N 5.54, S 12.68; Found: C 52.22, H 7.21, N 5.51, S 12.66.

12-(benzo[*d*]thiazol-2-ylthio)-*N,N*-bis(2-hydroxyethyl)-*N*-methyldodecan-1-aminium bromide (13d)

Yield: 62%. M.p. 106-107℃. IR(cm^{-1}): 3297(br), 2924(m), 2847(m), 1492(s), 1463(s), 1419(s), 1372(s), 1310(s), 1076(s), 1050(m), 1003(m), 882(s), 773(s),733(s), 663(s). ^{1}H NMR (400 MHz, DMSO-d^6) δ 8.03 - 7.31 (m, 4H, C_6H_5), 5.24 (t, J = 4.7 Hz, 2H, -OH), 3.81 (d, J = 3.7 Hz, 4H, O-CH_2), 3.44 (d, J = 2.0 Hz, 4H, N-CH_2), 3.36 (d, J = 3.5 Hz, 2H, N-CH_2), 3.34 - 3.24 (m, 2H, S-CH_2), 3.08 (s, 3H, N-CH_3), 1.75 (dt, J = 14.6, 7.2 Hz, 2H, -CH_2), 1.66 (s, 2H, -CH_2), 1.40 (d, J = 6.8 Hz, 2H, -CH_2), 1.24 (s, 14H, -CH_2). ^{13}C NMR (100 MHz, DMSO-d^6) δ 167.23, 153.19, 134.88, 126.76, 124.80, 122.19, 121.46, 63.55, 62.80, 55.17, 49.40, 33.24, 29.33, 29.28, 29.24, 29.16, 28.94, 28.86, 28.42, 26.21, 22.00.

Elemental analysis (%) calcd. for $C_{24}H_{41}BrN_2O_2S_2$ (531.92): C 54.02, H 7.74, N 5.25, S 12.02; Found: C 54.06, H 7.66, N 5.22, S 12.12.

Synthesis and characterization of compounds 16a-d

2-Mercapto-5-methyl-1,3,4-thiadiazole (1.32 g, 10 mmol), 1,6-dibromohexane (12.20 g, 50 mmol) or 1,8-dibromoctane (13.60 g, 50 mmol) or 1,10-dibromodecane (15.00 g, 50 mmol) or 1,12-dibromododecane (16.40 g, 50 mmol) and K_2CO_3 (6.9 g, 50 mmol) were mixture in 100 mL acetonitrile. The mixture was stirred for 3 h at room temperature (monitored by TLC). After filtrated, the filtrate was concentrated and the residue was purified on silica gel by column using petroleum ether/EtOAc (4:1, v/v) as the eluent to afford intermediate **15**. And excess dibromine alkyl was recycled. Intermediate and MDEA (1.19 g, 10 mmol) were mixture in 15 mL DMF. The mixture was stirred for 6 h at 40ºC (monitored by TLC). After added into 200 mL diethyl ether, the mixture was kept at -20ºC overnight, and then filtrated. The residue was recrystallized from methanol and diethyl ether to afford the colorless crystals **16a-d**.

***N*,*N*-bis(2-hydroxyethyl)-*N*-methyl-6-((5-methyl-1,3,4-thiadiazol-2-yl)thio)hexan-1-aminium bromide (16a)**

Yield: 58%. M.p. 56-57℃. IR(cm^{-1}): 3278(br), 2928(m), 2858(m), 1635(s), 1485(s), 1467(s), 1434(s), 1372(s), 1193(s), 1102(s), 1065(s), 1036(s), 985(s), 933(s), 820(s), 729(s), 605(m). ^1H NMR (400 MHz, DMSO-d^6) δ 5.24 (t, J = 4.8 Hz, 2H, -OH), 3.81 (d, J = 3.2 Hz, 4H, O-CH_2), 3.44 (s, 4H, N-CH_2), 3.31 (d, J = 13.4 Hz, 2H, N-CH_2), 3.26 (t, J = 7.1 Hz, 2H, S-CH_2), 3.08 (s, 3H, N-CH_3), 2.68 (s, 3H, -CH_3), 1.73 (dt, J = 14.9, 7.3 Hz, 4H, -CH_2), 1.44 (dt, J = 14.5, 7.4 Hz, 2H, -CH_2), 1.36 - 1.21 (m, 2H, -CH_2). ^{13}C NMR (100 MHz, DMSO-d^6) δ 165.65, 165.51, 63.58, 62.67, 55.17, 49.41, 33.82, 28.86, 27.80, 25.58, 21.82, 15.65. Elemental analysis (%) calcd. for $C_{14}H_{28}BrN_3O_2S_2$ (412.92): C 40.57, H 6.81, N 10.14, S 15.47; Found: C 40.51, H 6.69, N 10.20, S 15.52.

***N*,*N*-bis(2-hydroxyethyl)-*N*-methyl-8-((5-methyl-1,3,4-thiadiazol-2-yl)thio)octan-1-aminium bromide (16b)**

Yield: 52%. M.p. 74-75℃. IR(cm^{-1}): 3296(br), 2922(m), 2854(m), 1461(s), 1383(s), 1189(s), 1050(s), 964(s), 906(w), 856(s), 726(s), 604(m), 550(m). ^1H NMR (400 MHz, DMSO-d^6) δ 5.24 (t, J = 4.7 Hz, 2H, -OH), 3.81 (d, J = 3.4 Hz, 4H, O-CH_2), 3.45 (s, 4H, N-CH_2), 3.34 (d,

J = 5.9 Hz, 2H, N-CH_2), 3.25 (t, J = 7.1 Hz, 2H, S-CH_2), 3.09 (s, 3H, N-CH_3), 2.67 (s, 3H, -CH_3), 1.75 - 1.60 (m, 4H, -CH_2), 1.38 (s, 2H, -CH_2), 1.27 (d, J = 18.0 Hz, 6H, -CH_2). ^{13}C NMR (100 MHz, DMSO-d^6) δ 165.63, 165.54, 63.59, 62.83, 55.17, 49.43, 34.09, 29.11, 28.70, 28.56, 28.22, 26.09, 21.96, 15.66. Elemental analysis (%) calcd. for $C_{16}H_{32}BrN_3O_2S_2$ (442.5): C 43.43, H 7.29, N 9.50, S 14.49; Found: C 43.52, H 7.38, N 9.68, S 14.12.

***N,N*-bis(2-hydroxyethyl)-*N*-methyl-10-((5-methyl-1,3,4-thiadiazol-2-yl)thio)decan-1-aminium bromide (16c)**

Yield: 48%. M.p. 77-78℃. IR(cm^{-1}): 3304(br), 2931(m), 2851(m), 1463(s), 1419(s), 1379(s), 1350(m), 1295(s), 1237(s), 1193(s), 1080(s), 1018(m), 985(s), 915(s), 860(s), 798(s), 729(s), 638(s), 605(m). ^{1}H NMR (400 MHz, DMSO-d^6) δ 5.25 (t, J = 4.7 Hz, 2H, -OH), 3.82 (d, J = 3.4 Hz, 4H, O-CH_2), 3.45 (s, 4H, N-CH_2), 3.37 (s, 2H, N-CH_2), 3.25 (t, J = 7.1 Hz, 2H, S-CH_2), 3.08 (s, 3H, N-CH_3), 2.68 (s, 3H, -CH_3), 1.75 - 1.63 (m, 4H, -CH_2), 1.39 (s, 2H, -CH_2), 1.27 (s, 10H, -CH_2). ^{13}C NMR (100 MHz, DMSO-d^6) δ 165.24, 165.17, 63.21, 62.46, 54.79, 49.03, 33.71, 28.77, 28.75, 28.71, 28.46, 28.40, 27.95, 25.79, 21.60, 15.24. Elemental analysis (%) calcd. for $C_{18}H_{36}BrN_3O_2S_2$ (468.92): C 45.95, H 7.71, N 8.93, S 13.63; Found: C 45.86, H 7.68, N 8.87, S 13.72.

***N,N*-bis(2-hydroxyethyl)-*N*-methyl-12-((5-methyl-1,3,4-thiadiazol-2-yl)thio)dodecan-1-aminium bromide (16d)**

Yield: 46%. M.p. 95-96℃. IR(cm^{-1}): 3297(br), 2920(m), 2855(m), 1482(s), 1468(s), 1431(s), 1386(s), 1230(s), 1181(s), 1082(s), 1040(m), 964 (s), 932(m), 906(s), 883(s), 856(m), 797(s), 752(s), 726(s), 610(m), 550(m). ^{1}H NMR (400 MHz, DMSO-d^6) δ 5.25 (t, J = 4.8 Hz, 2H, -OH), 3.81 (d, J = 3.9 Hz, 4H, O-CH_2), 3.46 (t, J = 10.4 Hz, 4H, N-CH_2), 3.37 (d, J = 13.0 Hz, 2H, N-CH_2), 3.23 (t, J = 7.2 Hz, 2H, S-CH_2), 3.08 (s, 3H, N-CH_3), 2.67 (s, 3H, -CH_3), 1.69 (dt, J = 14.2, 7.0 Hz, 4H, -CH_2), 1.37 (s, 2H, -CH_2), 1.24 (s, 14H, -CH_2). ^{13}C NMR (100 MHz, DMSO-d^6) δ 165.60, 165.56, 63.60, 62.85, 55.18, 49.42, 34.12, 29.30, 29.27, 29.21, 29.16, 28.91, 28.83, 28.35, 26.21, 22.01, 15.63. Elemental analysis (%) calcd. for $C_{20}H_{40}BrN_3O_2S_2$ (498.6): C 48.18, H 8.09, N 8.43, S 12.86; Found: C 48.06, H 8.12, N 8.57, S 12.75.

Determination of antimicrobial activity

Different strains of bacteria and fungi which were representative in clinical practice and

agriculture were used as target pathogens in this study, including three strains of Gram-positive bacteria (*S. Aureus, α-H-tococcus*, *β-H-tococcus*), three strains of Gram-negative bacteria (*E. coli, P. aeruginosa*, *Proteus vulgaris*) and four strains of fungi (*Canidia albicans*, *Cytospora mandshurica, Physalospora piricola, Aspergillus niger*). The antimicrobial tests were assayed, according to standard CLSI methods for antimicrobial dilution susceptibility tests to measure the minimum inhibitory concentration (MIC) and minimum bactericidal concentration (MBC)[2].

MIC Assay

A broth tube dilution method was followed to determine the MIC values for all the screened compounds against the micro-organisms, and for comparison benzalkonium chloride (BZK) and chlorhexidine acetate (CA) was used as a reference. For sample preparation, each of the test compounds and reference were dissolved in DMSO or sterile water at a concentration of 1600 μg/mL, and further dilutions of the compounds and reference in culture medium MHB (Muller-Hinton Broth) were prepared at the required quantities of 400, 200, 100 etc., down to 0.39 μg/mL. Bacterial strains and fungal strains were maintained on MHA (Muller-Hinton Agar) medium at 37℃ or 28 ℃ for 12 h or 72h, respectively. The microbial inocula were prepared by suspension in 10 mL of culture medium for colonies from culture on MHA. The cell density of each inoculum was adjusted in culture medium of a 0.5 McFarland standard (1-2×10 ^{8} CFU/mL). The final amount applied was 10^5 CFU/mL for bacteria and fungi. The microbial inocula were added to the twofold diluted samples. One growth control tube was prepared with the addition of the compound and one blank tube was prepared without the addition of the microorganism. MIC values were read after incubation at 37℃ or 28 ℃ for bacterial and fungal strains, respectively. After 12 h or 72h, the last tube with no growth of micro-organisms was recorded to represent the MIC value expressed in μg/ml. All experiments were carried out three times.

MBC Assay

For MBC measurements, 100 μL × 3 (of each of the dilutions that showed no visible growth) was placed on agar plates in triplicate, and incubated at 37℃ or 28 ℃ for 12 h or 72h. The number of colonies on each plate was read, and the MBC was determined as the lowest concentration that achieved a 99.9% bactericidal in the agar plate.

Cytotoxicity Testing with MTT Method

Human immortalized epidermal cells (HaCat) line were cultured in Dulbecco's modified Eagle's medium containing 10% fetal bovine serum, 1% penicillin, and 1% streptomycin at 37°C in a 5% CO_2/95% air incubator MCO-15AC (SANYO). Human normal liver cells (LO2) line were cultured in Roswell Park Memorial Institute medium containing 10% fetal bovine serum, 1% penicillin, and 1% streptomycin at 37°C in a 5% CO_2/95% air incubator MCO-15AC (SANYO). HaCat or LO2 cells in 96-well plate were adjusted to 1.0 × 10^5 cells mL^{-1} and cells were passed and cell supernatant was pour out before plating on glass slide at 37°C, 5% CO_2 for 24 h. Cells were pretreated with 100 μL QAS for 24 h and cell supernatant with QAS was pour out and washed with PBS buffer system. Then, the cells were treated with the MTT solution (MTT, 3-(4,5-dimethythiazol-2-yl)-2,5-diphenyl-tetrazolium bromide, 20 μL, 5 mg/mL) for 4 h. The dark blue formazan crystal formed in the intact cells was solubilized with dimethyl sulfoxide, and absorbance at 570 nm was measured with a microplate reader (Multiskan MK3-Thermo Labsystems, USA). The results were expressed as percentage of the control value from the normal cells without treatments of QAS.

Hemolysis assay[3]

The human blood from healthy individual was collected in a tube containing heparin, and the blood was centrifuged at 1500 rpm for 3 min. The supernatant (Erythrocyte) was collected and plasma was discarded and further washed five times with sterile normal saline solution. Then the cells were diluted to 0.2% of their volume with normal saline solution. 2 mL of the diluted red blood cells suspension was mixed with (a) 2 mL of normal salinesolution as a negative control; (b) 2 mL of distilled water as a positive control; (c) 2 mL of QAS solution which were dispersed into normal saline solution at the different concentrations ranging from 3.125 to 400 μg/mL. Then the mixtures were shaken well and incubated for 1 h at 37 ℃. Finally, the mixtures were centrifuged for 5min at 2000 r/min and the absorbances of supernatant at 540 nm was measured by a microplate reader (Multiskan MK3-Thermo Labsystems, USA). The hemolysis ratio was calculated as follows: The hemolysis ratio(%) = $(OD_{Sample} - OD_{Negative})/(OD_{Positive} - OD_{Negative}) \times 100\%$. The hemolysis evaluation of QASs was performed in triplicate and results were expressed as the mean ± SD.

SEM images

After contacting compound **7d** with the concentration 100 μg/mL for 4 h and 12 h, respectively, the bacteria of *E. coli* and *S. Aureus* were collected by centrifuging at 7000 r/min for 5 min, and then fixed in 2.5% glutaraldehyde in phosphate buffer (0.2 mol/L, pH 7.2) at 4 °C for 4 h. After fixation, cells were washed three times with phosphate buffer (1ml, each time for 15 minutes) respectively, the samples of bacteria were then dehydrated for 15 min in increasing concentrations of aqueous acetone solution (30%, 50%, 70%, 80%, 90%, 100% (V/V)). These samples of bacteria were dried at CO_2 critical dryer, then gold-coated in a Quorum Q150R S Sputter coater. Finally, morphology of the bacterial cells was examined by EVO LS15 scanning electron microscopy (SEM, ZEISS, Germany).

TEM images

After contacting compound **7d** with the concentration 100 μg/mL for 4 h and 12 h, respectively, the bacteria of *E. coli* and *S. Aureus* were collected by centrifuging at 7000 r/min for 5 min, and then fixed in fresh 2.5% glutaraldehyde for at least 4 hrs at 4 °C, post-fixed in 1% osmium tetroxide for 1.5 hrs, dehydrated in a gradient ethanol series and infiltrated with Epon812. The samples were then embedded and cured at 37 °C for 12 hrs, 45 °C for 12 hrs and 60 °C for 24 hrs. Ultrathin sections were cut using Leica EM UC7 (Leica, Wetzlar, Germany) and stained with uranyl acetate and lead citrate before observation under the JEM-1400 transmission electron microscope (TEM; JEOL Ltd., Tokyo, Japan).

References

1. Tripathi P, Pal A, Jancik V, et al. Metal-assisted transformation of *N*-benzoyldithiocarbazate to 5-phenyl-1,3,4-oxadiazole-2-thiol in the presence of ethylenediamine, and its first row transition metal complexes. Polyhedron 2007;26:2597-2602.
2. Wang CH, Xie XR, Liu WS, et al. Quaternary ammonium salts substituted by 5-phenyl-1,3,4-oxadiazole-2-thiol as novel antibacterial agents with low cytotoxicity. Chemical Biology & Drug Design 2017; DOI: 10.1111/cbdd.13020.
3. Li J, Guo YY. Basic evaluation of typical nanoporous silica nanoparticles in being drug carrier: Structure, wettability and hemolysis. Materials Science and Engineering C 2017;73:670-673.
